# HER2 deficiency causes a developmental disorder with growth retardation and craniofacial malformations

**DOI:** 10.1172/JCI199043

**Published:** 2026-04-30

**Authors:** Huaxiang Zhao, Pan Wang, Yuhua Jiao, Huimei Huang, Min Yu, Qing He, Chengkai Pan, Shuang Guo, Wenbin Huang, Yunfei Jia, Qianying Kong, Huifang Peng, Yandong Han, Yuxia Hou, Zhanping Ren, Yongwei Tao, Fei Huang, Hongwei Jiang, Shan Sun, Yanying Dong, Jiuxiang Lin, Chunyan Yin, Xuechen Zhu, Feng Chen, Yi Ding

**Affiliations:** 1 Key Laboratory of Shaanxi Province for Craniofacial Precision Medicine Research, College of Stomatology, Xi’an Jiaotong University, Xi’an, Shaanxi, China.; 2 Department of Transfusion Medicine, The Second Affiliated Hospital of Xi’an Jiaotong University, Xi’an, Shaanxi, China.; 3 Department of Orthodontics, Peking University School and Hospital of Stomatology, Beijing, China.; 4 Department of Orthodontics, College of Stomatology, Xi’an Jiaotong University, Xi’an, Shaanxi, China.; 5 Research Center for Industries of the Future, Westlake University, Institute of Biology, Westlake Institute for Advanced Study, Hangzhou, Zhejiang, China.; 6 Westlake Laboratory of Life Sciences and Biomedicine, Hangzhou, Zhejiang, China.; 7 Department of Nephrology, Xi’an Children’s Hospital, The Affiliated Children’s Hospital, Xi’an Jiaotong University, Xi’an, Shaanxi, China.; 8 Department of Prosthodontics, Peking University School and Hospital of Stomatology, Beijing, China.; 9 National Center of Stomatology, National Clinical Research Center for Oral Diseases, National Engineering Laboratory for Digital and Material Technology of Stomatology, Beijing Key Laboratory for Digital Stomatology, Research Center of Engineering and Technology for Computerized Dentistry Ministry of Health, NMPA Key Laboratory for Dental Materials, Beijing, China.; 10 Department of Physiology and Pathophysiology, School of Basic Medical Sciences, Health Science Center, Xi’an Jiaotong University, Xi’an, Shaanxi, China.; 11 Department of Pediatrics, The Second Affiliated Hospital of Xi’an Jiaotong University, Xi’an, Shaanxi, China.; 12 Department of Orthodontics, Stomatological Center, Peking University Shenzhen Hospital, Shenzhen, Guangdong, China.; 13 Henan Key Laboratory of Rare Diseases, Endocrinology and Metabolism Center, The First Affiliated Hospital, and College of Clinical Medicine of Henan University of Science and Technology, Luoyang, Henan, China.; 14 Department of Cleft Palate-Craniofacial Surgery, College of Stomatology, Xi’an Jiaotong University, Xi’an, Shaanxi, China.; 15 Central Laboratory, Fujian Key Laboratory of Precision Medicine for Cancer, Key Laboratory of Radiation Biology of Fujian Higher Education Institutions, The First Affiliated Hospital, Fujian Medical University, Fuzhou, Fujian, China.; 16 Central Laboratory, National Regional Medical Center, Binhai Campus of the First Affiliated Hospital, Fujian Medical University, Fuzhou, Fujian, China.; 17 State Key Laboratory of Membrane Biology, Beijing Frontier Research Center for Biological Structure, School of Life Sciences, Tsinghua University, Beijing, China.; 18 State Key Laboratory of Female Fertility Promotion, Department of Human Anatomy, Histology and Embryology, School of Basic Medical Sciences, Peking University Health Science Center, Beijing, China.; 19 Department of Pediatrics, Children’s Medical Center, Peking University First Hospital, Beijing, China.; 20 Neuroscience Research Institute, Peking University, Beijing, China.; 21 Central Laboratory, Peking University School and Hospital of Stomatology, Beijing, China.

**Keywords:** Development, Genetics, Genetic diseases

## Abstract

The human epidermal growth factor receptor 2 (HER2) is a major therapeutic target in cancer. While the oncogenic effects of HER2 hyperactivation are well characterized, the biological consequences of its deficiency remain poorly defined. Here, through exome sequencing analyses of a cohort of 720 families affected by isolated or syndromic orofacial clefts, we unexpectedly identified 5 distinct rare germline *HER2* variants in 5 unrelated families with growth deficits, orofacial clefts, and other craniofacial, skeletal, and auditory anomalies. In *Xenopus* embryos, these variants failed to recapitulate the developmental effects of WT HER2. In cultured cells, they disrupted HER2 protein stability, membrane localization, or site-specific phosphorylation, resulting in diminished ERK signaling. Strikingly, knock-in mice expressing a patient-derived *HER2* variant and mice maternally exposed to Tucatinib, a recently approved anti-HER2 drug, both replicated patient phenotypes: delayed growth and diverse craniofacial abnormalities, including ocular dysgenesis, short jaws, and cleft palate. Collectively, our findings define a developmental disorder that we designate GRACE syndrome (Growth Retardation and Craniofacial Malformations Caused by HER2 Deficiency), establish HER2’s essential role in human growth and craniofacial morphogenesis, and reveal that HER2-targeted therapies during pregnancy can induce craniofacial defects and lifelong growth impairment in fetuses.

## Abstract 2

HER2 overactivity is well known to cause cancer. Unexpectedly, loss of HER2 function instead slows growth and deforms the face and skull.

## Graphical abstract

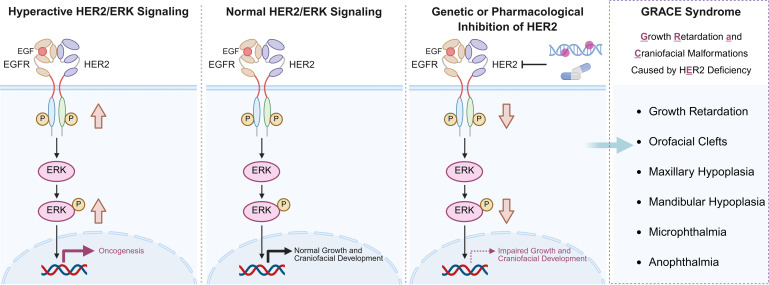

## Introduction

HER2 (also known as ERBB2) is a member of the epidermal growth factor receptor (EGFR) family of receptor tyrosine kinases. Upon activation through heterodimerization with other EGFR family members following ligand stimulation, HER2 initiates multiple downstream signaling cascades, including the ERK pathway, to regulate cell proliferation, migration, and differentiation (1). While HER2 overactivation is a well-established oncogenic driver (2, 3), the physiological effects of its deactivation are far less defined. In mice, HER2 loss disrupted cardiac and peripheral nervous system development, but its potential roles in later developmental stages have been masked by midgestational lethality caused by cardiac defects (4–6). Cardiac-rescued *Her2* knockout mice exhibited partial embryonic lethality and complete neonatal mortality with reduced body size, hinting at a role of HER2 in prenatal growth (7, 8). Further supporting this, limb cartilage- and bone-specific expression of Herstatin (an endogenous HER2 inhibitory splice variant) in mice resulted in shortened long bones before, but not after, embryonic day (E) 18.5, due to impaired proliferation of growth plate chondrocytes (9). Human congenital disorders associated with HER2 inactivation are exceptionally rare, with only a single documented case affecting the gastrointestinal tract (10). Here, we demonstrate that HER2 deficiency causes a developmental disorder that manifests as growth delay and craniofacial malformations in mice and humans.

## Results

### Rare HER2 variants in patients with growth delay and orofacial clefts.

**Figure 1:**
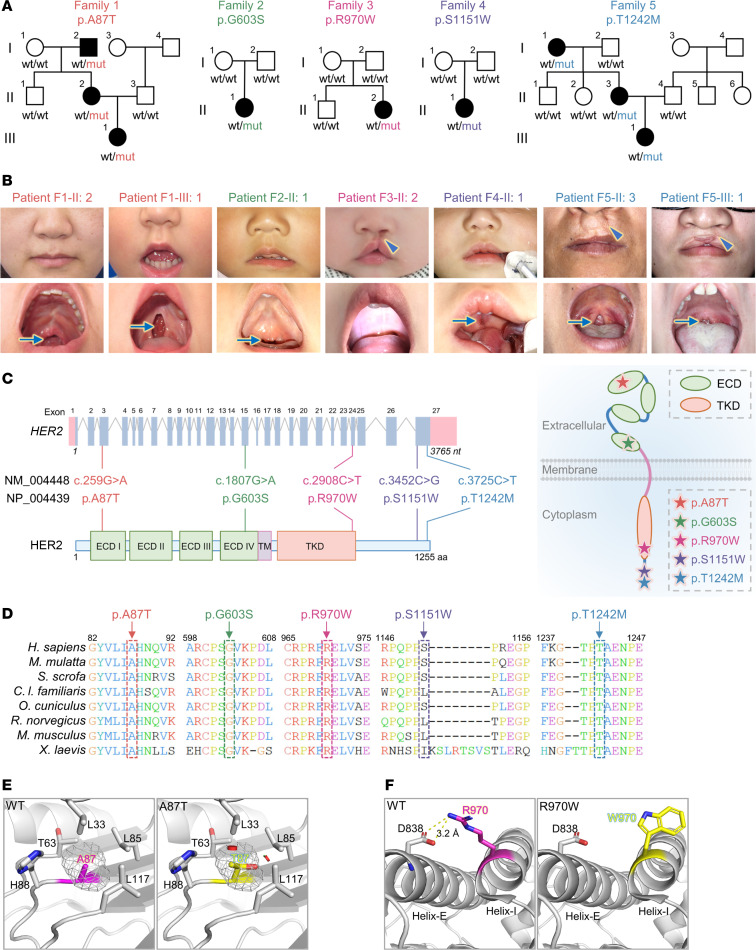
Heterozygous missense *HER2* variants cause growth impairment and craniofacial malformations. (**A**) Pedigrees of 5 families. Heterozygous *HER2* variants and genotypes of available family members are indicated. Squares denote male family members, circles female family members, open symbols unaffected, and black symbols affected. mut, mutant. (**B**) Images of affected family members. Arrowheads mark cleft lip (top row), and arrows denote cleft palate (bottom row). For patient F3-II:2, lip and palate images were obtained at the ages of 1 year and 5 years, respectively. (**C**) Genomic and protein locations of *HER2* variants (left) and the topological feature of HER2 in the plasma membrane (right). Variants are represented with stars. HER2 is comprised of an extracellular domain (ECD) with 4 subdomains (I, II, III, and IV), a single transmembrane domain (TM), and a cytoplasmic tyrosine kinase domain (TKD) followed by a C-terminal tail. (**D**) Protein sequence alignment of HER2 across various vertebrate species reveals the conservation of amino acids affected by the variants. Ser1150 is less conserved, while all other residues are highly conserved. (**E**) Structural modeling of the p.A87T variant. Ala87 is located on the C-terminal side of the parallel β-sheet in the extracellular domain I. It is buried within a hydrophobic cavity formed by Leu33, Leu85, Leu117, Thr63, and His88. Substituting alanine with threonine introduces a larger side chain containing a polar hydroxyl group, which may cause steric clashes (indicated by red disks) and disrupt the local hydrophobic environment. (**F**) Structural modeling of the p.R970W variant. Arg970 is positioned on the N-terminal side of Helix-I in the tyrosine kinase domain. Its positively charged side chain forms a salt bridge with Asp838 (indicated by yellow dashed lines). Substitution with tryptophan, which lacks a charged side chain, is predicted to disrupt this electrostatic interaction, potentially destabilizing the kinase domain.

We evaluated a cohort of 720 families (53 multiplex and 667 small families) affected by isolated orofacial clefts or clefts with additional congenital anomalies using exome sequencing. Among them, we identified 5 families (2 multiplex and 3 simplex families) exhibiting growth impairment, orofacial clefts, and other craniofacial, skeletal, and auditory abnormalities (Figure 1, A and B, and Table 1). Unexpectedly, analyses of exome sequences did not yield pathogenic variants in known genes associated with genetic syndromes presenting with these clinical manifestations, but instead converged on *HER2* as the potential causal gene on the basis of its predicted damaging scores (Table 1). In Families 1 and 5, 2 distinct monoallelic *HER2* missense variants (p.A87T and p.T1242M) segregated dominantly with the phenotype (Figure 1, A and B, Table 1, and Supplemental Figure 1; supplemental material available online with this article; https://doi.org/10.1172/JCI199043DS1). In Families 2–4, we detected 3 different de novo monoallelic *HER2* missense variants (p.G603S, p.R970W, and p.S1151W) (Figure 1, A and B and Table 1). All 5 *HER2* variants were verified by Sanger sequencing and were either absent or ultrarare (minor allele frequency < 0.0001) in the general population (Table 1, Supplemental Figure 2, and Supplemental Table 1). Mapping the disease-linked variants onto the HER2 structure revealed that these alleles are distributed across 3 functional domains: Ala87 and Gly603 in the extracellular domain, Arg970 in the tyrosine kinase domain, and Ser1151 and Thr1242 in the cytoplasmic tail (Figure 1C). Sequence alignment of human HER2 with its orthologues revealed high evolutionary conservation at Ala87, Gly603, Arg970, and Thr1242, whereas Ser1151 displayed lower conservation (Figure 1D). While high-confidence predictions or explicit structural resolution for regions involving residues Gly603, Ser1151, and Thr1242 are lacking, structural modelling suggested that p.A87T and p.R970W variants might disrupt local structure and thus function of HER2 (Figure 1, E and F).

These findings suggest that rare *HER2* variants may represent the genetic cause of a human developmental disorder characterized by growth delay, craniofacial malformations, and an array of associated symptoms.

### Attenuation of HER2/ERK signaling by patient variants.

**Figure 2:**
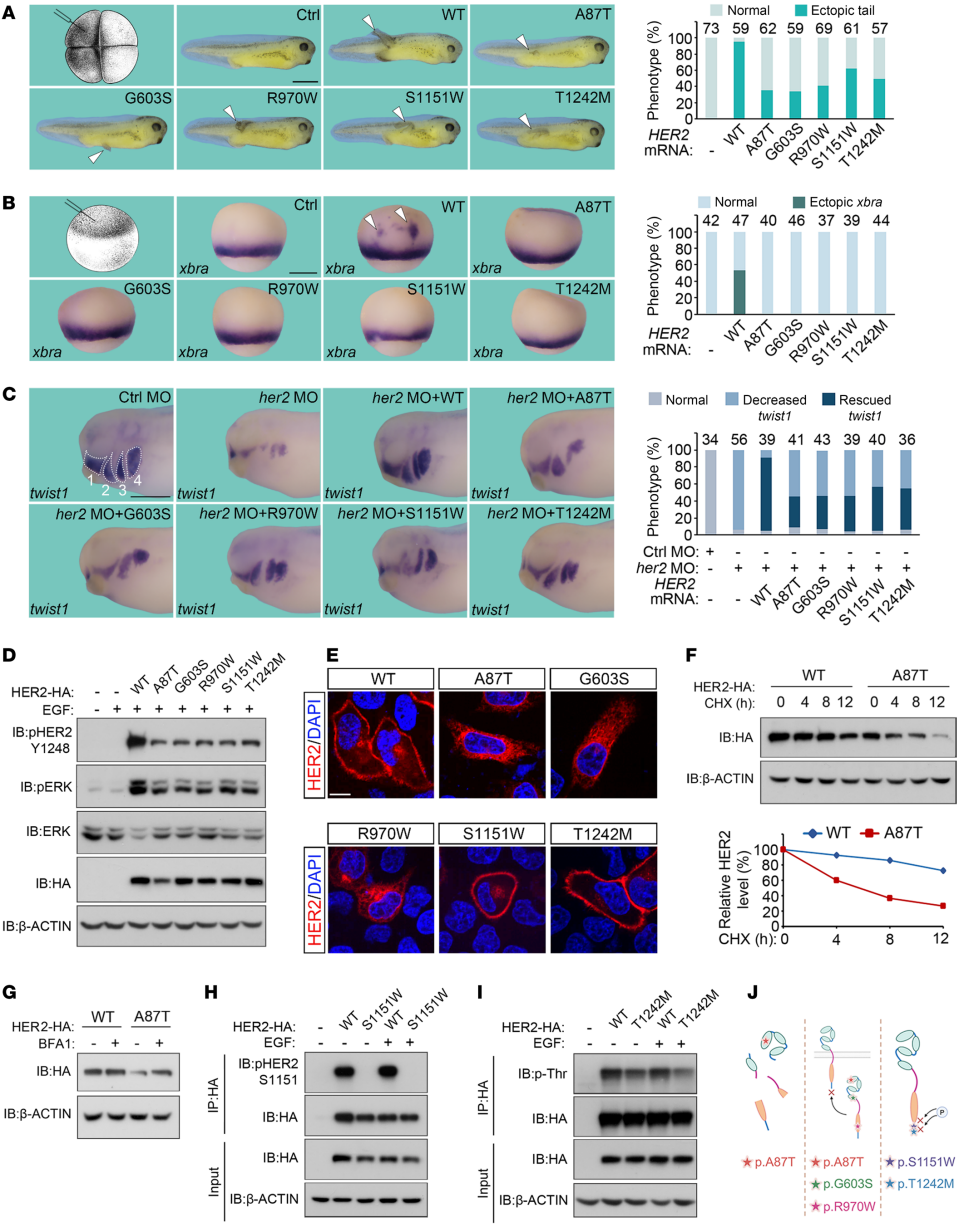
Patient variants impair HER2/ERK signaling. (**A**) HER2 variants are less effective than WT HER2 in inducing the formation of supernumerary tails. Arrowheads indicate extra tails. Lateral view, anterior to right. Scale bar: 1 mm. *Xenopus* images were reproduced from *Development* (35). (**B**) WT, but not mutant HER2 induces ectopic *xbra* expression in the ectoderm. Arrowheads indicate ectopic *xbra* signals in the ectoderm. Lateral view, animal pole to top. Scale bar: 0.5 mm. *Xenopus* images were reproduced from *Development* (35). (**C**) HER2 variants are less efficient than WT HER2 in rescuing impaired *twist1* expression in *her2*-depleted embryos. *twist1* expression was detected in the 4 streams of CNCCs migrating towards the pharyngeal arches (marked by white dashed circles): the mandibular (no. 1), hyoid (no. 2), anterior branchial (no. 3), and posterior branchial (no. 4) streams. Lateral view, anterior to left. Scale bar: 0.5 mm. (**D**) Patient variants reduce HER2 and ERK activities. *HER2* knockout HEK293T cells were transfected, stimulated with EGF, and harvested for immunoblot analysis. (**E**) p.A87T, p.G603S, and p.R970W variants disrupt HER2 membrane localization. Hela cells were transfected and processed for immunofluorescent analysis. Scale bar: 10 μm. (**F**) p.A87T HER2 shows shortened half life. *HER2* knockout HEK293T cells were transfected, treated with cycloheximide (CHX), and harvested for immunoblot analysis. The quantified HER2 levels normalized to β-ACTIN are at the bottom. (**G**) Bafilomycin A1 (BFA1) partially normalizes p.A87T HER2 expression level. *HER2* knockout HEK293T cells were transfected, treated with BFA1, and harvested for immunoblot analysis. (**H**) p.S1151W variant abolishes HER2 phosphorylation at Ser1151. *HER2* knockout HEK293T cells were transfected, stimulated with EGF, and harvested for immunoprecipitation (IP) and immunoblot analysis. (**I**) p.T1242M HER2 displays diminished threonine phosphorylation. The experiment was performed as in panel **H**, except that the p.T1242M variant was used. (**J**) Summary of the pathogenic mechanisms of *HER2* variants.

To investigate the pathogenicity of the *HER2* variants, we first assessed the signaling potential of these *HER2* variants in *Xenopus* embryos. We synthesized mRNAs encoding WT or mutant human HER2 and found that the p.A87T variant was expressed at lower levels than WT HER2 or other mutants (Supplemental Figure 3A). In line with a previous study (11), overexpression of WT HER2 potently induced formation of supernumerary tails in 94.9% of embryos by the tailbud stage, a phenotype indicative of ERK pathway activation (12, 13). In contrast, all HER2 variants displayed markedly reduced capacity to elicit this phenotype (Figure 2A). Additionally, overexpression of WT HER2, but not the variants, induced ectopic *xbra* (a mesoderm marker and ERK cascade target) expression in the ectoderm at stage 10.5, with *xbra* levels in the circumferential layer of mesoderm cells unchanged (Figure 2B). Her2 is required for cranial neural crest cell (CNCC) induction and migration in *Xenopus* (14). At the tailbud stage, the impaired CNCC induction and migration in *her2*-depleted embryos, visualized by *twist1* expression in the pharyngeal arches, was largely rescued by WT HER2 overexpression but only partially by the HER2 variants (Figure 2C and Supplemental Figure 3B). Therefore, the patient-derived *HER2* variants behave as loss-of-function alleles that are likely to attenuate HER2/ERK signaling in *Xenopus* embryos.

We next examined the impact of these variants on HER2/ERK signaling and explored their pathogenic mechanisms in cultured cells. When overexpressed in *HER2* knockout HEK293T cells, all 5 HER2 variants showed reduced autophosphorylation at Tyr1248 upon EGF stimulation, a readout of HER2 kinase activity (1, 15, 16), and a diminished capacity to induce ERK phosphorylation relative to WT HER2 (Figure 2D and Supplemental Figure 4). Membrane localization is a prerequisite for HER2 signaling activity (1). Upon ectopic expression in Hela cells, WT HER2, and the p.S1151W and p.T1242M variants displayed strong membrane localization, whereas p.A87T, p.G603S, and p.R970W variants were mislocalized to the cytoplasm (Figure 2E). Notably, consistent with the results in *Xenopus* embryos, the p.A87T variant exhibited clearly lower expression levels compared with WT HER2, pointing to compromised protein stability (Figure 2D). This was confirmed by cycloheximide chase assay that clearly demonstrated a reduced half life of p.A87T HER2 (Figure 2F). Moreover, the reduced expression level of p.A87T HER2 could be partially restored following a 10-hour treatment with the lysosome inhibitor Bafilomycin A1 (BFA1), arguing that this variant is intrinsically unstable and targeted to lysosome for degradation (Figure 2G). PFKFB3 (6-Phosphofructo-2-Kinase/Fructose-2,6-Biphosphatase 3)-induced HER2 phosphorylation at Ser1151 has been shown to be required for its kinase activity (Supplemental Figure 4D) (17, 18). Consistently, a phospho-specific HER2 antibody targeting Ser1151 showed a complete loss of signal for p.S1151W variant compared with WT HER2 (Figure 2H). Since Thr1242 has also been identified as a phosphorylation site (19), we tested whether p.T1242M variant could disrupt Thr1242 phosphorylation and thus impair HER2 kinase activity. As expected, a phospho-threonine antibody showed a weaker signal for the p.T1242M variant compared with WT HER2 (Figure 2I). Thus, the patient-derived *HER2* variants are bona fide loss-of-function alleles that cannot sustain proper HER2/ERK signaling and impair protein function through different mechanisms: the p.A87T, p.G603S, and p.R970W variants disrupt membrane localization; the p.A87T variant additionally undermines protein stability; and the p.S1151W and p.T1242M variants abolish site-specific phosphorylation (Figure 2J). Together, these results from cultured cells demonstrate that the patient-derived *HER2* variants are bona fide loss-of-function alleles that dampen HER2/ERK signaling.

Based on the functional evidence from *Xenopus* embryos and mammalian cells establishing loss-of-function effects, and following the ACMG-AMP guidelines (American College of Medical Genetics and Genomics and the Association for Molecular Pathology) (20), we classified these 5 *HER2* variants as pathogenic (Supplemental Table 2).

### Growth delay and craniofacial anomalies in Her2 knock-in mice.

**Figure 3:**
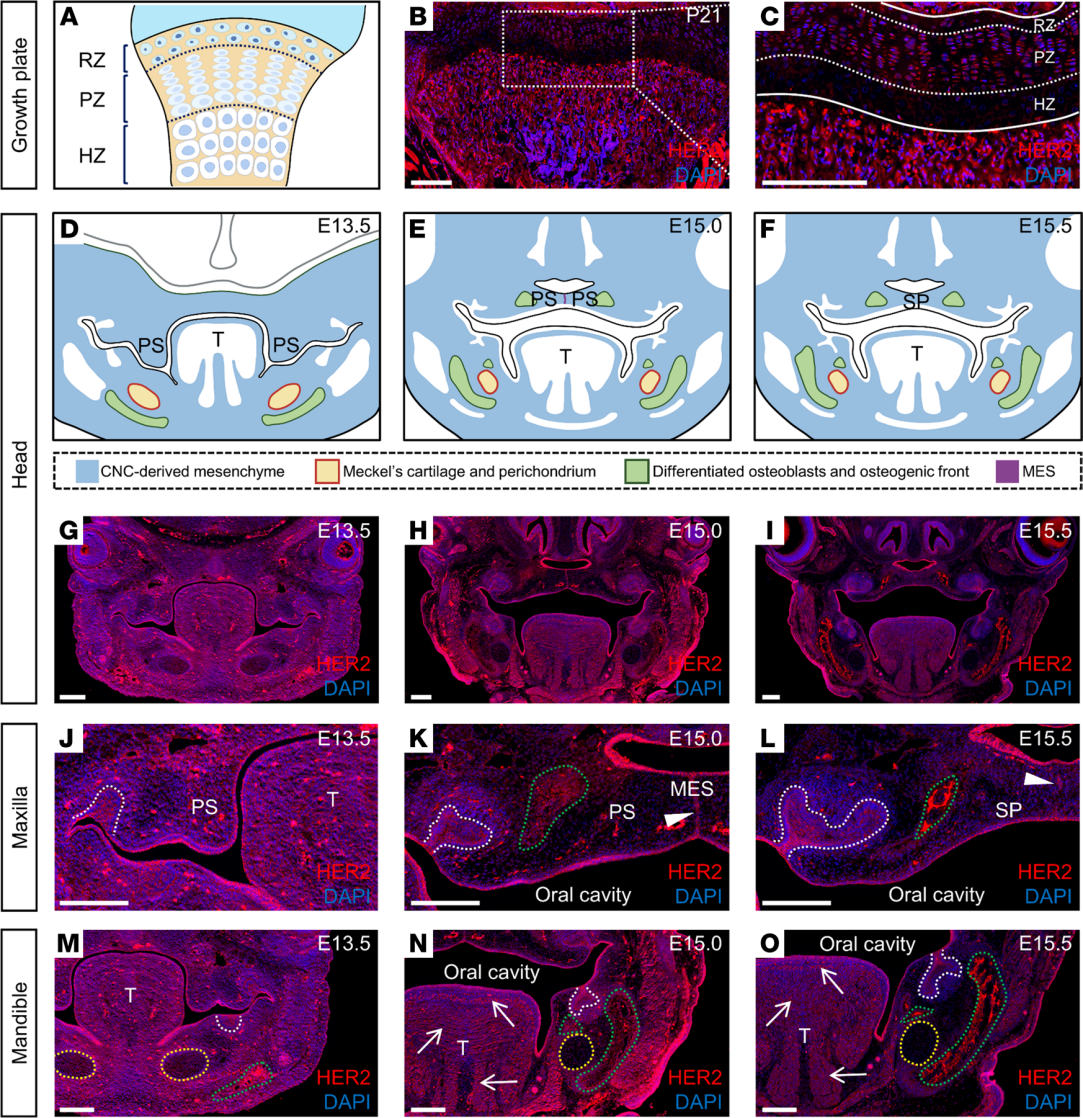
HER2 is expressed in growth plate proliferating chondrocytes and craniofacial tissues in mice. (**A**) Schematic diagram of mouse growth plate. RZ, resting zone; PZ, proliferating zone; HZ, hypertrophic zone. (**B** and **C**) Immunostaining of HER2 (red) in the P21 mouse proximal tibial growth plate shows its predominant expression in proliferating chondrocytes. The boxed region in panel **B** is shown at higher magnification in panel **C**. (**D**–**F**) Schematic diagrams of coronal sections of mouse craniofacial region at indicated stages (36, 37). (**G**–**I**) Overview of HER2 immunostaining (red) in coronal sections of mouse heads at E13.5, E15.0, and E15.5. Aside from epidermis and oral epithelium, HER2 is also expressed in the eyes and cranial neural crest–derived (CNC-derived) mesenchyme. (**J**–**L**) Magnified views of the maxillary region from panels **G**–**I**. HER2 shows strong expression in the differentiated osteoblasts and osteogenic front (green dashed circles in panels **K** and **L**) and the tooth germs (white dashed lines in panels **J**–**L**). In addition, HER2 expression is detected in midline epithelial seam formed by the fusing palatal shelves at E15.0 and diminishes as the seam underwent degradation at E15.5 (white arrowheads in panels **K** and **L**). (**M**–**O**) Magnified views of the mandibular region from panels **G**–**I**. Strong HER2 expression is detected in differentiated osteoblasts and osteogenic front (green dashed circles in panels **M**–**O**), but not in Meckel’s cartilage and perichondrium (yellow dashed circles in panels **M**–**O**). HER2 expression is also observed in the mandibular tooth germs (white dashed lines in panels **M**–**O**) and in the tongue muscle (white arrows in panels **N** and **O**). T, tongue; PS, palatal shelf; SP, secondary palate; MES, midline epithelial seam; CNC, cranial neural crest. Scale bar: 200 μm in all panels.

To delineate the potential roles of HER2 in mammalian growth and craniofacial development, we analyzed its spatiotemporal expression patterns in murine growth plates and embryonic craniofacial tissues. HER2 exhibited predominant localization to proliferating chondrocytes within the growth plate at postnatal day 21 (P21), consistent with its expression pattern observed during embryonic stages (9) (Figure 3, A–C). In craniofacial structures, robust HER2 expression was observed in CNC-derived mesenchyme, ocular primordia, osteogenic lineages of the maxillary and mandibular prominences, and the midline epithelial seam during palatal shelf fusion (Figure 3, D–O). These results suggest that HER2 may play a pleiotropic role in mammalian longitudinal bone growth and craniofacial morphogenesis.

To model the pathogenic effects of *HER2* variants, we generated a knock-in mouse line harboring the patient-derived p.A87T variant, selected for its detrimental effects on HER2 membrane localization and stability (Supplemental Figure 5, A and B). While heterozygous *Her2* p.A87T knock-in mice were healthy and viable, homozygous mutant mice exhibited incomplete preweaning lethality by P21, indicating that the p.A87T allele is partially lethal (Supplemental Figure 5C). To investigate whether embryonic lethality contributed to the partial postnatal mortality, we performed timed mating and analyzed embryonic survival at key gestational stages. At E13.5, the observed mutant embryo ratio conformed well to the expected Mendelian distribution. Strikingly, by E18.5, the proportion of recovered mutant embryos plummeted to 17.3% (119 of 688), a substantial deviation from the anticipated 25%, indicating that approximately 30% of mutant embryos succumbed between E13.5 and E18.5 (Supplemental Figure 5C).

**Figure 4:**
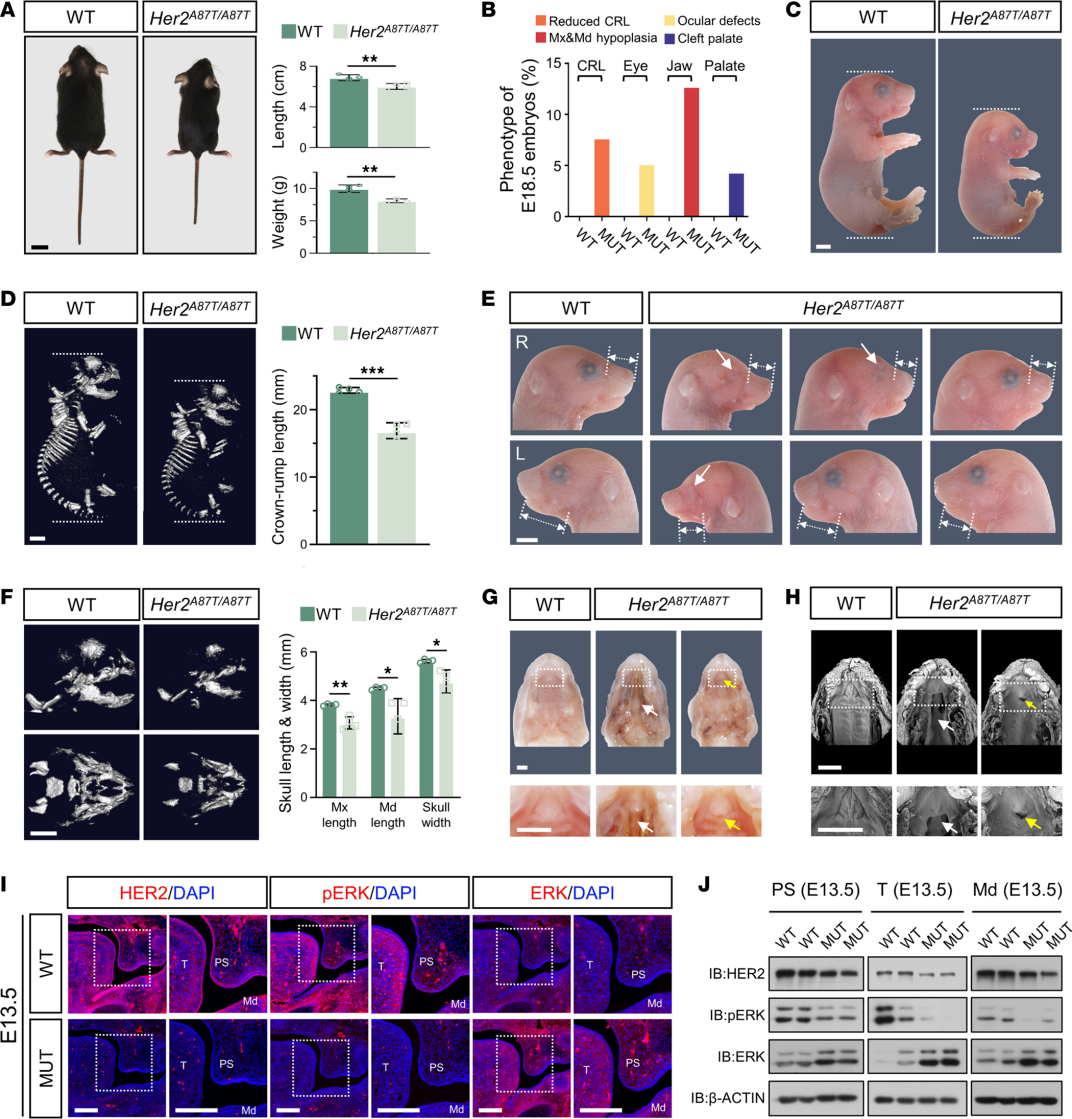
*Her2* p.A87T knock-in mice exhibit growth deficits and craniofacial anomalies. (**A**) *Her2^A87T/A87T^* mice display reduced body length and weight at P21. Representative images (left; scale bar: 1 cm) and quantification (right; *n* = 4 per genotype; **P < 0.01) are shown. (**B**) Penetrance of developmental defects in E18.5 *Her2^A87T/A87T^* embryos, including reduced crown-rump length (CRL), ocular defects, maxillary (Mx) and mandibular (Md) hypoplasia, and cleft palate. MUT, mutant. (**C** and **D**) *Her2^A87T/A87T^* embryos (7.56%, 9 of 119) display reduced crown-rump length. Representative stereomicroscope images (**C**) and microcomputed tomography images (**D**) of E18.5 embryos (scale bar: 2 mm) and quantification (*n* = 4 per genotype; ***P < 0.001) are shown. (**E**) *Her2^A87T/A87T^* embryos exhibit maxillary and mandibular hypoplasia (12.6%, 15 of 119) and ocular defects (anophthalmia, 5.04%, 6 of 119) at E18.5. Dashed lines with double arrowheads indicate maxillary (top) and mandibular (bottom) lengths. Arrows indicate missing eyes. Each column corresponds to the same embryo. R, right view; L, left view. Scale bar: 2 mm. (**F**) Microcomputed tomography images of E18.5 craniofacial skeleton confirming reduced maxillary and mandibular lengths and narrower skull width in *Her2^A87T/A87T^* embryos. Scale bar: 2 mm. Quantification is shown (*n* = 4 per genotype; *P < 0.05, **P < 0.01). (**G**) *Her2^A87T/A87T^* embryos (4.2%, 5 of 119) exhibit cleft palate at E18.5. Boxed regions are magnified in the bottom. Arrows indicate complete (middle) or incomplete (right) cleft palate. Scale bar: 1 mm. (**H**) Representative scanning electron micrographs confirming the cleft palate in *Her2^A87T/A87T^* embryos shown in panel **G**. Scale bar: 1 mm. (**I** and **J**) Immunofluorescence (**I**) and immunoblot (**J**) showing reduced HER2 and phospho-ERK (pERK) signals, but unchanged ERK level, in the palate shelf (PS), mandible (Md), and tongue (T) of E13.5 *Her2^A87T/A87T^* embryos. Boxed regions in panel **I** are magnified on the right. Scale bar: 200 μm. MUT, mutant. Unpaired *t* tests (**A**, **D**, and **F**).

We next characterized the developmental phenotypes in the surviving mutant embryos and juvenile mice. At P21, a portion of surviving mutant mice (22.7%, 5 of 22) displayed pronounced reduction in both body size and body weight, replicating the growth delay observed in the patients (Figure 4A). Among the surviving mutant embryos at E18.5, 7.6% (9 of 119) of them exhibited intrauterine growth restriction, as evidenced by the severe reduction in crown-rump length (Figure 4, B–D). Moreover, the mutant embryos presented a spectrum of craniofacial anomalies, including ocular defect (anophthalmia, 5.0%, 6 of 119), maxillary and mandibular hypoplasia (12.6%, 15 of 119), and cleft palate (4.2%, 5 of 119) (Figure 4, B and E–H, Supplemental Figure 6, and Supplemental Figure 7A). In line with the normal neurological and cognitive findings in our patients (Table 1), we observed no significant differences in cognitive performance among WT, heterozygous, and homozygous *Her2* p.A87T knock-in mice (Supplemental Figure 8).

The prenatal growth delay and craniofacial anomalies in *Her2* p.A87T knock-in mice show substantial overlap with those reported in ERK-deficient mice (21, 22). Combined with our cell culture results, these findings imply that HER2 likely mediates its effects predominantly via the ERK pathway during these developmental processes. Indeed, a marked allele-dosage–dependent reduction in HER2 expression and ERK phosphorylation was evident in the craniofacial region of mutant embryos at E13.5 (Figure 4I). In addition, palatal, tongue, and mandibular tissues in mutant mice all exhibited a marked decline in HER2 expression and ERK phosphorylation relative to their WT littermates at E13.5 (Figure 4J).

Taken together, these findings in *Her2* p.A87T knock-in mice unveil that HER2 deficiency directly induces prenatal and postnatal growth delay and diverse craniofacial anomalies, mediated through diminished HER2/ERK signaling.

### Growth delay and craniofacial anomalies induced by maternal exposure to Tucatinib in mice.

**Figure 5:**
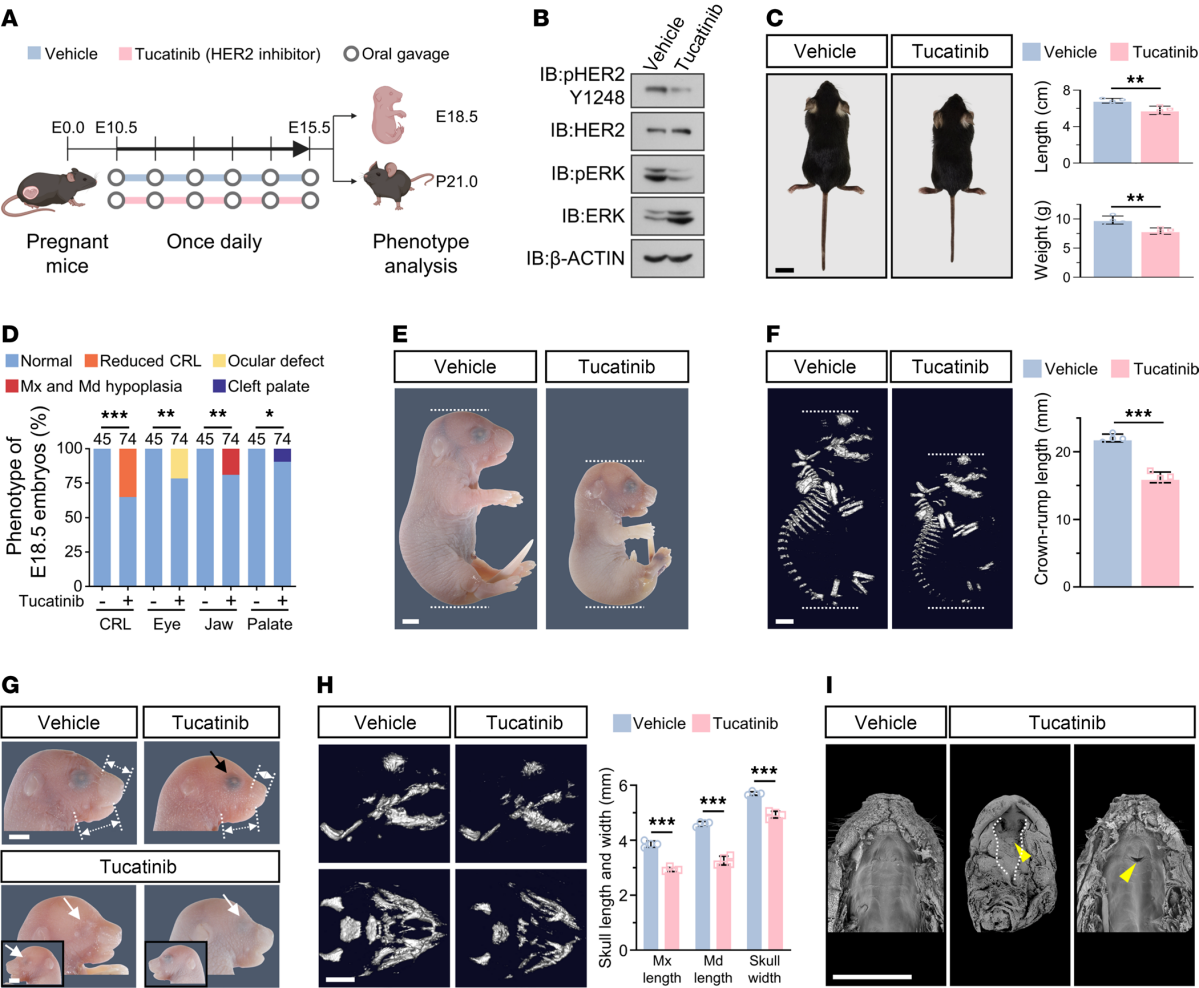
Maternal exposure to Tucatinib results in growth deficits and craniofacial anomalies in mice. (**A**) Schematic of Tucatinib administration. (**B**) Immunoblot showing reduced HER2 and pERK levels in E15.5 palate shelves following Tucatinib treatment. (**C**) Mice exposed to Tucatinib exhibit reduced body length and weight at P21. Representative images (left; scale bar: 1 cm) and quantification (right; *n* = 4 per group; **P < 0.01) are shown. (**D**) Penetrance of developmental defects in E18.5 embryos exposed to Tucatinib, including reduced crown-rump length (CRL), ocular defects, maxillary (Mx) and mandibular (Md) hypoplasia, and cleft palate. *P < 0.05, **P < 0.01, ***P < 0.001. (**E** and **F**) Tucatinib-treated embryos (35.14%, 26 of 74) display reduced crown-rump length. Representative stereomicroscope images (**E**) and microcomputed tomography images (**F**) of E18.5 mouse embryos (scale bar: 2 mm) treated with vehicle or Tucatinib and quantification (*n* = 4 per group; ***P < 0.001) are shown. (**G**) Tucatinib-treated embryos exhibit ocular defects (microphthalmia, 12.16%, 9 of 74; anophthalmia, 9.46%, 7 of 74) and maxillary and mandibular hypoplasia (18.92%, 14 of 74) at E18.5. Dashed lines with double arrowheads indicate maxillary and mandible lengths. Black arrow, microphthalmic eye; white arrows, missing eyes. Insets show the contralateral eye. Scale bar: 2 mm. (**H**) Microcomputed tomography images of E18.5 craniofacial skeletons confirming reduced maxillary and mandibular lengths and narrower skull width following Tucatinib exposure. Scale bar: 2 mm. Quantification (right; *n* = 4 per group; ***P < 0.001) is shown. (**I**) Tucatinib treatment induces cleft palate (9.5%, 7 of 74) in E18.5 embryos. Scanning electron micrographs show complete (middle) or incomplete (right) cleft palate. Yellow arrowheads indicate cleft; dashed lines mark unfused palate shelves. Scale bar: 2 mm. Unpaired *t* tests (**C**, **F**, and **H**) and χ^2^ tests (**D**).

To further substantiate the causal link between HER2 deficiency and human growth delay and craniofacial malformations, we evaluated the embryonic and postnatal developmental outcomes of pharmacological inhibition of HER2 in mice. Tucatinib is an oral, HER2-specific small-molecule tyrosine kinase inhibitor recently approved for the treatment of previously treated HER2-positive metastatic breast cancer and colorectal cancer (23–25). To bypass the midgestational lethality caused by cardiac defects, as observed in *Her2-*null embryos (6), we administered Tucatinib orally once daily to pregnant WT C57BL/6J dams from E10.5, the developmental stage at which *Her2*-null embryos die, until E15.5. Offspring were assessed at P21 to evaluate body size and body weight, or dams were sacrificed at E18.5 to analyze fetal outcomes (Figure 5A).

While Tucatinib treatment was well tolerated by the pregnant dams, it potently suppressed HER2 and ERK activities in the offspring (Figure 5B). Remarkably, at P21, Tucatinib treatment led to significant reduction in both body size and body weight in 41.2% (14 of 34) of the offspring (Figure 5C). At E18.5, 35.1% (26 of 74) of embryos prenatally exposed to Tucatinib displayed significantly reduced crown-rump length, confirming intrauterine growth restriction (Figure 5, D–F). In addition, maternal exposure to Tucatinib also resulted in a wide range of craniofacial anomalies in offspring, including ocular defects (microphthalmia, 12.2%, 9 of 74; anophthalmia, 9.5%, 7 of 74), maxillary and mandibular hypoplasia (18.9%, 14 of 74), and cleft palate (9.5%, 7 of 74) (Figure 5, D and G–I, and Supplemental Figure 7B). These defects were virtually indistinguishable from those in *Her2* p.A87T knock-in mice, but they occurred at a notably higher frequency, presumably due to the circumvention of early embryonic lethality.

Collectively, the replication of both prenatal and postnatal growth delay and craniofacial anomalies by maternal exposure to Tucatinib in mice offers unequivocal evidence that HER2 deficiency acts as a key driver for these developmental defects.

## Discussion

Our study establishes HER2 deficiency as the direct cause of a developmental disorder that we name GRACE syndrome (Growth Retardation and Craniofacial Malformations Caused by HER2 Deficiency). Both loss-of-function *HER2* variants (genetic factor) and maternal exposure to anti-HER2 agents (environmental factor) contribute to the incidence and, consequently, the prevalence of this condition. In striking contrast to HER2’s canonical oncogenic paradigm, this condition unveils its fundamental role in human development, exposing an underappreciated aspect of HER2 biology.

Our work uncovers HER2’s essential function in human growth regulation. Previous murine studies have demonstrated that HER2 loss leads to prenatal growth impairment (8, 9). We now establish loss-of-function *HER2* variants as a genetic cause of growth failure, providing definitive evidence for HER2’s direct role in human growth control. These findings suggest that HER2 deficiency may underlie a subset of human growth disorders currently classified as “idiopathic”.

Our results unmask an unexpected role of HER2 in CNCC-driven craniofacial morphogenesis. To date, the only human congenital disorder linked to HER2 inactivation is visceral neuropathy-2 (VSCN2; OMIM 619465), resulting from the p.A710V loss-of-function variant and manifesting as gastrointestinal dysmotility (10). Intriguingly, while orofacial clefts stem from defects in cranial neural crest development, VSCN2 arises from deficits in vagal neural crest development (26–30), collectively demonstrating a general role of HER2 in neural crest biology and suggesting that HER2 deficiency may represent an etiological nexus for neurocristopathies with undetermined genetic origin.

Breast cancer complicates approximately 1 in 3,000 pregnancies, with rising incidence rates paralleling the trend of increasing maternal age observed in recent decades (31). Anti-HER2 agents have become the standard of care for HER2-positive breast cancer, and their use during pregnancy, though rare, is expected to become more common (32). Clinical studies have linked HER2-targeted therapies during pregnancy to specific severe fetal and neonatal complications, including oligohydramnios, congenital respiratory tract disorders, and neonatal renal failure (32). Here, we show that maternal Tucatinib exposure in mice recapitulates GRACE syndrome phenotypes, revealing previously unrecognized growth and craniofacial risks of HER2 inhibition during pregnancy. Hence, in cases where HER2-targeted therapies are deemed clinically necessary during pregnancy, rigorous monitoring of fetal craniofacial development and longitudinal tracking of prenatal and postnatal growth are imperative.

## Methods

### Sex as a biological variable.

Our study examined male and female animals, and sex was not considered as a biological variable.

### Patients.

We enrolled a total of 720 Chinese families, including 53 multiplex and 667 small families, affected by isolated orofacial clefts or clefts with additional congenital anomalies. All participants underwent systematic phenotyping, including detailed clinical evaluations for structural malformations and neurocognitive assessments. Standard deviation (SD) scores for body height and body weight were calculated using: (1) the Growth Standard for Children under 7 Years of Age (WS/T 423-2022) for pediatric participants, and (2) reference data from China’s Fifth National Physical Fitness Monitoring Bulletin and Human diameters of Chinese adults (GB/T 10000-2023) for adults. Individuals with height measurements > 2 SD below the population mean for age and sex were classified as having growth delay.

### Exome sequencing.

Genomic DNA was isolated from patient peripheral blood samples using the QIAamp DNA Blood Mini Kit (Qiagen #51006). Exome sequencing was conducted on the BGISEQ-500 platform (BGI Inc., China) and the Illumina PE150 platform (Novogene Co., Ltd., China), with sequence reads aligned to the Human GRCh37/hg19 reference genome. Variants were prioritized and filtered according to standard protocols as described in our previous studies (33, 34). Potential causative variants were classified as pathogenic or likely pathogenic according to the ACMG-AMP guidelines. In all five families exhibiting growth impairment, orofacial clefts, and other congenital anomalies, *HER2* missense variants were identified as the top candidate. Primers used for PCR-Sanger sequencing verification are listed in Supplemental Table 1.

### Generation of the Her2 p.A87T knock-in mouse line.

The *Her2* p.A87T mouse line was generated at Shanghai Model Organisms Center Inc. using the CRISPR/Cas9 system and following standard protocols with the gRNA (GCAGTGGGACGTGTTTCACTCGG) and the single-stranded oligo donor DNA (GACATCCAGGAAGTCCAGGGATACATGCTCATCACTCACAATCGAGTGAAACACGTCCCACTGCAGAGGTTGCGCATCGTGAGAGGGACTCAGCTCTTTGAGGACAAGTATGCCCTGGCTGTGCTAGACAACCGAGACCCTTTGGACAACGTCACCACCGCCGCCCCAGGCAGAACCCCAGAAGGGCTGCGGGAGCTGCAGCTTCGAAGTCTCACAG). The offspring were genotyped by PCR Sanger sequencing. Mice with correct genotypes were backcrossed with C57BL/6J WT mice for at least three generations before being inbred breeding to generate homozygotes for phenotypic analysis.

### Tucatinib treatment in mice.

Tucatinib (MCE #HY-16069), a highly selective tyrosine kinase inhibitor of HER2, was dissolved in a solution composed of 5% DMSO, 40% PEG300, 5% Tween-80, and 50% 1XPBS, yielding a final concentration of 2 mg/ml. Pregnant C57BL/6J WT mice (Cyagen Biosciences Inc.) were administered orally with either Tucatinib (50 mg/kg) or the vehicle solution (lacking Tucatinib) once daily from E10.5 to E15.5. At E18.5, embryos were harvested for analysis, yielding 45 embryos from vehicle-treated dams and 74 embryos from Tucatinib-treated dams. In parallel, 47 vehicle-exposed offspring and 34 Tucatinib-exposed offspring were assessed for body size and weight at P21.

### Scanning electron microscopy.

After careful removal of mouse mandible and tongue, the palates were fixed in a solution containing 2% glutaraldehyde (GA) and 2% PFA in PBS. The samples were then dehydrated through a graded ethanol series, followed by critical point drying using CO_2_. Finally, the samples were sputter-coated with a thin gold layer. Images were captured and processed with a scanning electron microscope (Hitachi #TM-1000).

### Other methods.

The detailed experimental procedures for DNA constructs, structural modeling of *HER2* variants, cell culture and transfection, generation of the *HER2* knockout HEK293T cell line, immunoblotting of cultured cells, *Xenopus* embryos, and mouse tissues, immunoprecipitation, immunofluorescent staining, *Xenopus* embryo manipulations, mRNA synthesis and morpholinos, whole-mount in situ hybridization, *Xenopus* illustration, micro-computed tomography, and behavioral tests are provided in the Supplemental material.

### Statistics.

Samples were randomly assigned to experimental groups. Sample sizes for animal experiments are indicated in the figures or their legends. Biochemical experiments were performed with at least 3 independent replicates. Continuous variables are expressed as mean ± standard deviations, and categorical variables are reported as percentages (with frequencies). For comparisons of continuous variables between 2 groups, unpaired *t* tests were used, and for comparisons of categorical variables between 2 groups, χ^2^ tests were used. For comparisons of continuous variables among multiple groups, 1-way ANOVA or Kruskal-Wallis tests were used as appropriate. Statistical significance was set at *P* < 0.05.

### Study approval.

All work with patients was approved by the Ethics Committee in Hospital of Stomatology, Xi’an Jiaotong University (xjkqll[2019]NO.014) and Ethical Committee of Peking University Hospital of Stomatology (PKUSSIRB-201520012). Written informed consent was obtained from participants or their guardians for both sample collection and the use of photographs and records of consent have been retained.

All animal experiments involving *Xenopus laevis* and mice were approved by the Committee on the Ethics of Animal Experiments of Xi’an Jiaotong University (Approval No. XJTULAC2020-411) and the Peking University Animal Ethics Committee (Approval No. LA2018192). The experiments were conducted in strict accordance with established animal care and use guidelines.

### Data availability.

All data are available in the main text or the supplementary materials. Values for all data points in graphs are reported in the Supporting Data Values file. The identified *HER2* variants were deposited in the National Genomics Data Center (NGDC), China National Center for Bioinformation (CNCB) (GVM001248).

## Author contributions

Y Ding, HZ, FC, XZ, and CY designed the research; HZ, Y. Jiao, HH, MY, WH, Y. Jia, Y. Hou, ZR, YT, JL, and CY recruited clinical participants and conducted genetic experiments and analysis; Y Ding, HZ, Y. Jiao, QH, FH, Y Dong, and SS performed biochemical experiments; XZ, PW, QK, HP, and HJ performed Xenopus experiments; HZ, PW, Y. Jiao, HH, MY, QH, CP, SG, and Y. Han performed mouse experiments; Y Ding, HZ, FC, XZ, and CY analyzed the data; Y Ding, HZ, FC, XZ, and CY wrote the manuscript.

## Conflict of interest

The authors have declared that no conflict of interest exists.

## Funding support

- Beijing Natural Science Foundation (grant Z240020 to XZ).
- National Natural Science Foundation of China (grants 82572120 and 32070803 to Y Ding; grant 82170916 to FC; grant 32370859 to XZ; grant 82373594 to CY; grant 82370909 to Y. Hou; and grant 82001030 to HZ).
- Key Research and Development Project of the Shaanxi Province Health Scientific Research and Innovation Capacity Enhancement Program (grant 2025YF-05 to HZ).
- Shaanxi Provincial Youth Science and Technology Star Program (grant 2025ZC-KJXX-140 to HZ).
- IIT Clinical Research Fund of The Second Affiliated Hospital of Xi’an Jiaotong University (grant T-007 to CY).
- Fundamental Research Funds for the Peking University (grant PKU2022XGK001 to FC).
- National Key Research and Development Program of China (grant 2022YFA1206103 to FC).
- Peking University Medicine plus X Pilot Program-Platform Construction Project (grant 2024YXXLHPT012 to XZ).
- Clinical Medicine Plus X - Young Scholars Project of Peking University, and the Fundamental Research Funds for the Central Universities (grant PKU2024LCXQ005 to XZ).

## Supplementary Material

Supplemental data

Unedited blot and gel images

Supporting data values

## Figures and Tables

**Table 1:**
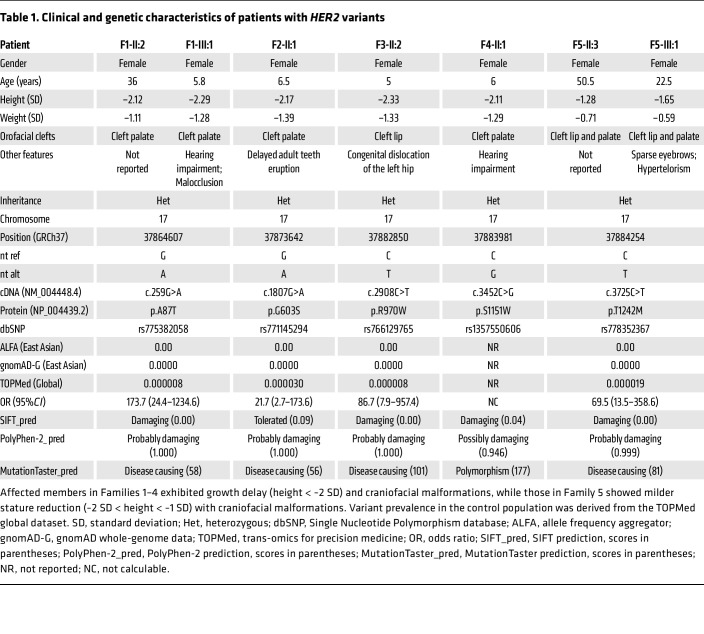
Clinical and genetic characteristics of patients with *HER2* variants

## Version 1 04/30/2026

In-Press Preview

## Version 2 06/15/2026

Electronic publication
