# Supplementary material for: HER2 deficiency causes a developmental disorder with growth retardation and craniofacial malformations
Source: J Clin Invest. 2026 Apr 30;136(12):e199043. doi: 10.1172/JCI199043 (PMC13262720; doi:10.1172/JCI199043)
Supplement: Supplemental data [file jci-136-199043-s196.pdf]

Supplemental material for

HER2 deficiency causes a developmental disorder with growth retardation and craniofacial malformations

Huaxiang Zhao[1,2,3,4†], Pan Wang[5,6,†], Yuhua Jiao[1,4,†], Huimei Huang[7,†], Min Yu[8,9], Qing He[10], Chengkai Pan[10], Shuang Guo[2], Wenbin Huang[11], Yunfei Jia[1,4], Qianying Kong[12], Huifang Peng[12], Yandong Han[1,4], Yuxia Hou[1,4], Zhanping Ren[1,13], Yongwei Tao[1,13], Fei Huang[14,15], Hongwei Jiang[12], Shan Sun[16], Yanying Dong[17], Jiuxiang Lin[3,9], Chunyan Yin[2]*, Xuechen Zhu[18,19,20]*, Feng Chen[9,21]*, Yi Ding[10,17]*

[†]These authors contributed equally to this work.

***Correspondence to:** yinchunyan@mail.xjtu.edu.cn (C. Yin); zhuxc@bjmu.edu.cn (X. Zhu); chenfeng2011@hsc.pku.edu.cn (F. Chen); dingyi1510@xjtu.edu.cn (Y. Ding)

The PDF file includes:

Material and Methods

References

Supplemental Figure 1-8

Supplemental Table 1-2

Materials and methods

DNA constructs

Human *HER2* cDNA (NM_004448) was a kind gift from Professor Yeguang Chen in Tsinghua University and was inserted into *pCS2* or *pmCherry-N1* vectors (1). Patient missense variants were introduced using the QuickMutation™ Site-directed Gene Mutagenesis Kit (Beyotime #D0206M).

Structural modeling of *HER2* variants

To evaluate the structural impact of the variants, mutant models were generated using the mutagenesis tool in PyMOL (Schrödinger, LLC, v2.5.0), following methodologies described in a previous study (2). Side chain rotamers were selected using the backbone-dependent rotamer library in PyMOL, which incorporates both rotamer probability and steric clash avoidance with surrounding atoms. Due to the absence of high-confidence predictions or explicit structural resolution for regions involving residues Gly603, Ser1151, and Thr1242, structural modeling was restricted to the p.A87T and p.R970W variants. The crystal structures of the extracellular domain (PDB: 1N8Z) and the tyrosine kinase domain (PDB: 3PP0) of WT HER2 were used as templates for the modeling.

Cell culture and transfection

HEK293T and Hela cell lines (both from National Infrastructure Cell Line Resource of China and tested negative for mycoplasma contamination) were cultured in DMEM (Cellmax #CGM101.05) supplemented with 10% fetal bovine serum (Omega, #FB-01), 2mM L-glutamine and 1% penicillin-streptomycin, in a 5% CO_2 incubator at 37°C.

Both cell lines were transfected with jetPRIME® reagent (Polyplus #101000046) following the manufacturer's instructions.

Generation of the *HER2* knockout HEK293T cell line

The *HER2* knockout HEK293T cell line was generated by Cyagen Biosciences Inc. using the CRISPR/Cas9 system with a gRNA targeting the sequence CAGAGGCTGCGGATTGTGCG, following a standard protocol. Single-cell clones were isolated, collected, and genotyped using PCR with the following primers: GGCCTTTGCTTTCACTGATGA (forward) and TTTTCCAATCTTCTGAGGGCGT (reverse). The results were validated by Sanger sequencing. Loss of HER2 protein expression was also confirmed by immunoblot analysis.

Immunoblotting of cultured cells, *Xenopus* embryos, and mouse tissues

To examine the effects of *HER2* variants on HER2 and ERK activities, *HER2* knockout HEK293T cells in 12 well plate were transfected with WT or mutant *pCS2 HER2-HA* constructs (0.5 μg). 30 hours after transfection, cells were washed with PBS twice and serum starved for 16 hours. Then cells were stimulated with 0.2 ng/ml EGF (R&D systems, #236-EG) for 10 min. For cycloheximide chase assay, *HER2* knockout HEK293T cells in 12 well plate were transfected with WT or *pCS2 HER2 A87T-HA* constructs (0.2 μg). 24 hours after transfection, cells were treated with 20 μg/ml cycloheximide (#HY-12320) for 0, 4, 8 and 12 hours. For Bafilomycin A1 (#HY-100558) treatment, *HER2* knockout HEK293T cells in 12 well plate were transfected with WT or *pCS2 HER2 A87T-HA* constructs (0.2 μg). 30 hours after transfection, cells were treated with 0.2 μM Bafilomycin A1 for 10 hours. Finally, cells treated as

described above were lysed in 150 ul TNE lysis buffer (50 mM Tris-HCl pH 7.4, 150 mM NaCl, 1 mM EDTA, 1% Nonidet P-40) supplemented with protease inhibitors (Beyotime #P1005) and phosphatase inhibitors (Beyotime #P1081). *Xenopus laevis* embryos expressing WT or mutant HER2-HA were collected and lysed using the same method as cultured cells. For mouse tissues, palatal shelves, tongues, and mandibles from WT or mutant mice were carefully dissected under a stereoscope, snap-frozen in liquid nitrogen, and homogenized in TNE lysis buffer containing the same inhibitors using a tissue lyser.

After lysis, the samples from cultured cells, *Xenopus laevis* embryos, and mouse tissues were centrifuged, and the supernatant was collected. Protein extracts were separated on 10% SDS–polyacrylamide gels and transferred to polyvinylidene difluoride (PVDF) membranes. Immunoblotting was performed using primary antibodies followed by horseradish peroxidase (HRP)-conjugated secondary antibodies (1:3000, Mouse-HRP, Jackson Immuno #71503510; 1:3000, Rabbit-HRP, Jackson Immuno #711035152). Membranes were then exposed to Fuji Medical X-ray Films (FUJIFILM #4741019274) and developed using a Developer and Fixer Kit (Beyotime #P0020). Primary antibodies used were: rabbit anti-HA (1:5000, Proteintech #51064-2-AP), mouse anti-β-actin (1:10000, Origene #TA811000), mouse anti-β-tubulin (1:10000, Origene #TA503129), rabbit anti-HER2 (1:2000, Proteintech #18299-1-AP), rabbit anti-phospho-HER2 (Tyr1248) (1:1000, Cell signaling #2247S), rabbit anti-phospho-HER2 (Ser1151) (1:3000, Abcepta #AP3781c), rabbit anti-phospho-ERK (1:3000, Cell signaling #9101S), rabbit anti-ERK (1:10000, Cell signaling #4695S),

mouse anti-phospho-Thr (1:3000, Cell signaling #9386S).

Immunoprecipitation

HER2 knockout HEK293T cells in 6 well plate were transfected with WT or mutant *pCS2 HER2-HA* (1.5 µg) constructs. 30 hours after transfection, cells were washed with PBS twice and serum starved for 16 hours. Then cells were stimulated with EGF (0.2 ng/ml, 10 min) and lysed in 400 µl of TNE lysis buffer supplemented with protease inhibitors and phosphatase inhibitors. Lysates were centrifuged at 12,000 rpm for 15 minutes at 4ºC to remove cell debris, and supernatants were collected. 20 µl of anti-HA Affinity Beads (Smart-Lifesciences #SA068005) were incubated with 350 µl of cell lysate overnight at 4 °C with head-over-head rotation. Beads were then washed in TNE lysis buffer for 5 times (10 min/time) and finally heated for 5 min at 95 °C in 50 µL of 2X Laemmli buffer to elute protein complexes, followed by analysis through SDS/PAGE and immunoblotting.

Immunofluorescent staining

Hela cells grown on 12 well plates containing glass coverslips (NEST #801011) were transfected with WT or mutant human *pmCherry-N1-HER2* constructs. 48 hours later, cells were washed twice with PBS (137 mM NaCl, 2.7 mM KCl, 10 mM Na_2HPO_4, 1.8 mM KH_2PO_4) to remove medium, fixed in 4% PFA (Sigma #P6148) in PBS for 20 min, and permeabilized with 0.2% Triton X-100 (Beyotime #P0096) in PBS for 10 min on ice. Next, cells were washed 3 times (10 min/time) with PBS and mounted onto glass slides in ProLong™ Gold Antifade Mountant with DAPI (Invitrogen #P36935) to stain cell nuclei. Images were photographed using a Nikon A1 confocal microscope and

analyzed with ImageJ software.

P21 mouse proximal tibia sections were deparaffinized, rehydrated, and subjected to antigen retrieval using Sodium Citrate Buffer (10 mM Sodium Citrate, 0.05% Tween 20, pH 6.0). Whole mouse embryos were fixed in 4% paraformaldehyde (PFA; Sigma #P6148) and processed into 4-μm-thick coronal paraffin sections for immunostaining. Primary antibodies used were: rabbit anti-HER2 (1:200, Cell signaling #2165S), rabbit anti-phospho-ERK (1:200, Cell signaling #4370S), rabbit anti-ERK (1:200, Cell signaling #4695S). The secondary antibody used was: Cy™3 AffiniPure™ Donkey Anti-Rabbit IgG (H+L) (1:200, Jackson ImmunoResearch Laboratories #711165152). Images were captured with a 3DHISTECH Pannoramic DESK P-MIDI P250 automated whole-slide fluorescence scanner and analyzed using SlideViewer software.

Xenopus embryo manipulations

Xenopus laevis were obtained from the Xenopus Resource Center (www.xenopus.cn). Embryos were obtained through *in vitro* fertilization and cultured in 0.1X Marc's Modified Ringers (100 mM NaCl, 2 mM KCl, 1 mM $MgSO_4 \cdot 7H_2O$, 2 mM $CaCl_2 \cdot 2H_2O$, 5 mM HEPES pH 7.4) and staged according to Zahn and colleagues (3).

mRNA synthesis, morpholinos, and _Xenopus_ embryos microinjection

For *in vitro* mRNA synthesis, WT or mutant human *pCS2-HER2-HA* were linearized with Not1 and transcribed with SP6 RNA polymerase using the mMESSAGE mMACHINE SP6 Transcription Kit (Thermo Scientific #AM1340). The previously described translation-blocking antisense morpholino oligonucleotides (MO) targeting *Xenopus laevis her2* gene was synthesized by Gene Tools. The sequences of the

morpholino: CAGCTCCATCATCTACTCCATGTCC (4).

To induce ectopic tails, *Xenopus* embryos were injected at the four-cell stage with mRNAs (500 pg) encoding WT or mutant human HER2 into one ventral-animal blastomere and analyzed at tailbud stage. To assess ectopic *xbra* expression in the ectoderm, *Xenopus* embryos were injected at the one-cell stage with mRNAs (400 pg) encoding WT or mutant human HER2 into the animal region and processed for whole-mount in situ hybridization for *xbra* at stage 10.5. For the rescue experiment, *Xenopus* embryos were injected at the eight-cell stage into one dorsal-animal blastomere with either control morpholino (Ctrl MO; 4 ng), *her2* MO (4 ng), or *her2* MO (4 ng) combined with mRNAs (500 pg) encoding WT or mutant human HER2, targeting the cranial neural crest, and processed for whole-mount in situ hybridization for *twist1* at stage 26. Quantification results for all experiments are shown on the right. The number of embryos per condition is indicated above each column.

Whole-mount in situ hybridization

Whole-mount in situ hybridization of *Xenopus laevis* embryos was performed as described at http://www.hhmi.ucla.edu/derobertis.

***Xenopus* illustration**

Xenopus laevis illustrations were adapted from Xenbase (www.xenbase.org RRID:SCR_003280) using Adobe Illustrator (3). The illustrations shown in Figure 2A and 2B are © Natalya Zahn (2022).

Micro-computed tomography

Mouse embryos at E18.5 were fixed in 4% PFA and scanned with a microcomputed

tomography scanner (PerkinElmer #Quantum GX) using the following parameters: pixel size 90 μm, voltage 90 kV, current 80 μA, scan time 4 min, and field of view 45 mm. The 3D images were reconstructed using 3DViewer software (PerkinElmer). To measure crown-rump length, mice from various genetic backgrounds or treatment groups were uniformly positioned, and the vertical distance from the parietal bone's apex to the caudal vertebra was recorded. To measure maxillary length, mandibular length, and skull width, mouse heads from different groups were consistently positioned. Maxillary length was defined as the distance from the most anterior point of the premaxilla to the posterior inferior point of the maxilla; mandibular length as the distance from the posterosuperior point of the condyle to the most anterior point of the lower mandibular margin; and skull width as the distance between the bilateral intersections of the zygoma and the zygomatic process of the temporal bone (Supplemental Figure 6).

Behavioral tests

Cognitive function was evaluated using the Y-maze, T-maze, and novel object recognition test (ORT) as previously described (5, 6). Nine- to ten-week-old male wild-type, heterozygous ($Her2^{+/A87T}$), and homozygous ($Her2^{A87T/A87T}$) mice were used ($n \geq 9$ per genotype). For the Y-maze test, mice were placed at the center of a three-arm apparatus and allowed to freely explore for 10 min. Movements were recorded, and spontaneous alternation (%) was calculated as (number of alternations / [total arm entries - 2]) × 100%. For the food-induced T-maze, mice were food-deprived for 24 h prior to testing. The apparatus consisted of one start arm and two goal arms, with a 2-g

food pellet placed in one goal arm during the training trial. Mice were habituated in the start arm for 20 s before free exploration. After locating and consuming the food, mice were returned to their home cages. Ten minutes later, a second trial was conducted without food, and the latency to reach the previously baited goal arm was recorded as an index of short-term memory. For the ORT, mice were habituated to an open-field arena and then exposed to two identical objects for 10 min. After a one-hour retention interval, one object was replaced with a novel object, and exploration was recorded for 10 min. Exploration was defined as nose-directed investigation within 2 cm of the object. The discrimination index was calculated as (T_novel - T_familiar) / (T_novel + T_familiar). Mice with total object exploration time < 20 s were excluded from analysis.

References

1. Huang F, et al. HER2/EGFR-AKT signaling switches tgfbeta from inhibiting cell proliferation to promoting cell migration in breast cancer. *Cancer Res.* 2018;78(21):6073-6085.
2. Yang Z, et al. A mutation-induced drug resistance database (MdrDB). *Commun Chem.* 2023;6(1):123.
3. Zahn N, et al. Normal table of xenopus development: a new graphical resource. *Development.* 2022;149(14):dev200356.
4. Mathavan K, et al. The ectodomain of cadherin-11 binds to erbB2 and stimulates Akt phosphorylation to promote cranial neural crest cell migration. *PLoS One.* 2017;12(11):e0188963.
5. Chen Y, et al. Synaptotagmin-11 deficiency mediates schizophrenia-like behaviors in mice via dopamine over-transmission. *Nat Commun.* 2024;15(1):10571.
6. Bevins RA, Besheer J. Object recognition in rats and mice: a one-trial non-matching-to-sample learning task to study 'recognition memory'. *Nat Protoc.* 2006;1(3):1306-1311.

Supplemental Figures

Total variants from exome sequencing					
Family 1			Family 5		
II-2 (affected mother)	II-3 (unaffected father)	III-1 (affected daughter)	II-3 (affected mother)	II-4 (unaffected father)	III-1 (affected daughter)
120,767	117,772	131,643	128,879	130,360	128,287

Variants with MAF < 5‰ in 1000 Genomes Project (Asian)					
Family 1			Family 5		
II-2 (affected mother)	II-3 (unaffected father)	III-1 (affected daughter)	II-3 (affected mother)	II-4 (unaffected father)	III-1 (affected daughter)
15,138	14,332	16,335	15,761	16,521	15,652

Coding and splicing variants					
Family 1			Family 5		
II-2 (affected mother)	II-3 (unaffected father)	III-1 (affected daughter)	II-3 (affected mother)	II-4 (unaffected father)	III-1 (affected daughter)
1,727	1,721	1,784	1,780	1,828	1,784

Dominant inheritance	
Family 1	Family 5
373	346

Predicted to be damaging by ≥ 2 tools	
Family 1	Family 5
226	203

Combined analysis of two multiplex families
ABI3BP, ANKS1A, BAGE3, HER2, ESRRA, MUC3A, MUC6, MUC16, NCOR1, OTOP1, PDE4DIP, SEC63, SYNE2, TARBP1, ZFHX3

Manual validation		
HER2	Family 1	Family 5
	c.259G>A / p.A87T	c.3725C>T / p.T1242M

Supplemental Figure 1. Summary of exome sequencing analysis for Families 1 and 5. Two independent missense variants in *HER2* were identified. The numbers indicate the count of variants.

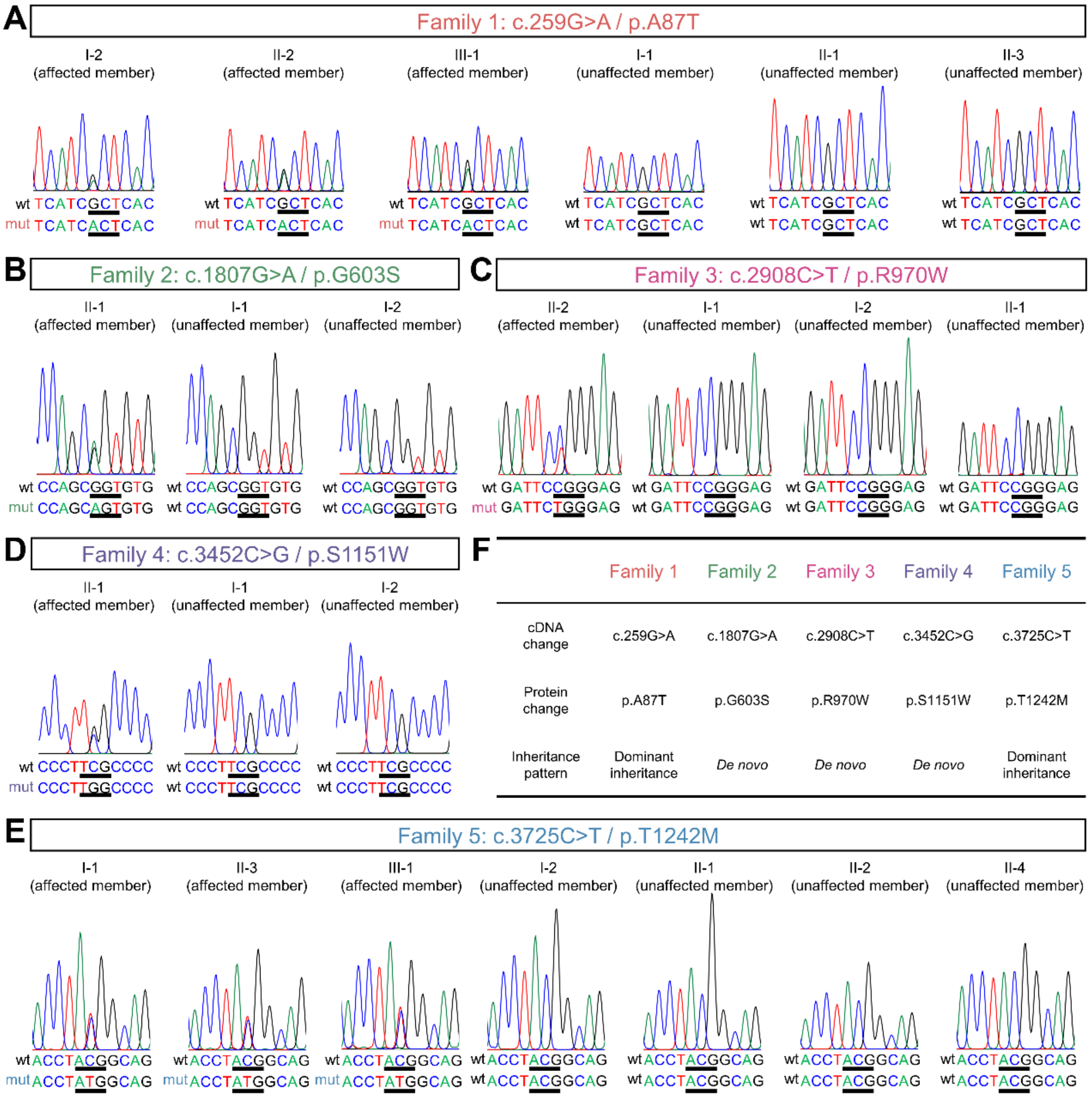

Supplemental Figure 2. Validation of *HER2* variants in the probands and available family members in Families 1-5 by Sanger sequencing. The p.A87T and p.T1242M variants were inherited in a dominant manner from the affected parents, whereas the p.G603S, p.R970W, and p.S1151W variants occurred *de novo*.

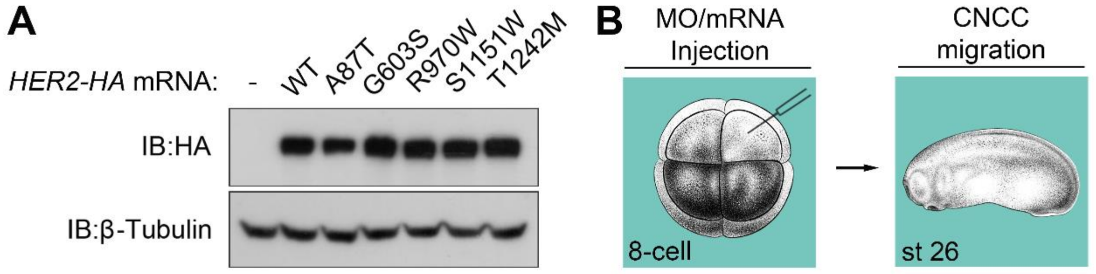

Supplemental Figure 3. Expression of mRNAs encoding WT or mutant human HER2 in *Xenopus* embryos. (A) Immunoblot analysis of HER2 in stage 10.5 *Xenopus* embryos injected at one-cell stage with mRNAs (500 pg) encoding WT or mutant human HER2. β-Tubulin served as a loading control. **(B)** Schematic diagrams of *Xenopus* embryos showing mRNA and/or morpholino (MO) injection into one dorsal-animal cell at eight-cell stage (left) to target the cranial neural crest and analysis of CNCC migration at stage (st) 26. *Xenopus* illustrations © Natalya Zahn (2022). Related to Figure 2C.

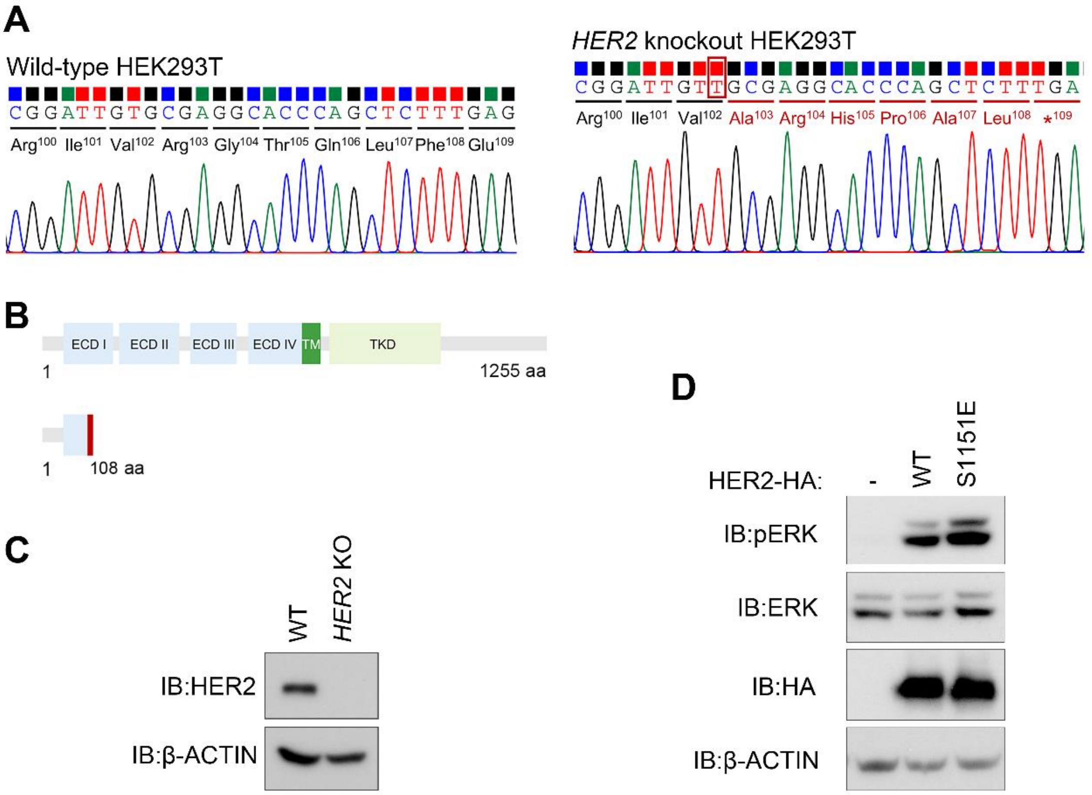

Supplemental Figure 4. Characterization of the *HER2* knockout HEK293T cell line and the effect of HER2 S1151E mutant. (A) Sanger sequencing revealed a homozygous thymidine insertion (highlighted by the red box) in codon 102, resulting in a stretch of six missense amino acids (codons 103–108) and a premature termination at codon 109, ultimately producing a truncated protein. **(B)** Graphical depiction of the WT and mutant HER2 proteins resulting from the homozygous thymidine insertion described in panel **A**. **(C)** Immunoblot analysis confirms the loss of HER2 protein expression in *HER2* knockout HEK293T cells compared to WT controls. β-ACTIN served as a loading control. (**D**) S1151 phosphorylation enhances HER2 activity. *HER2* knockout HEK293T cells were transfected with WT or p.S1151E HER2 and harvested for immunoblot analysis. β-ACTIN served as a loading control.

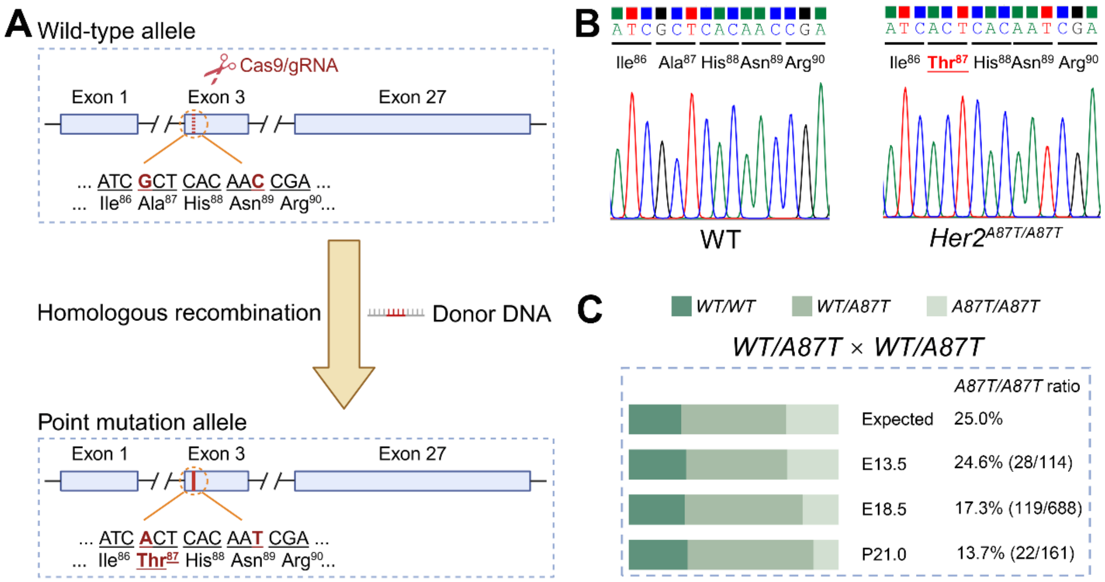

Supplemental Figure 5. *Her2* p.A87T knock-in mice show partial embryonic lethality and preweaning lethality. (A) Schematic diagram showing generation of *Her2* p.A87T knock-in mice by CRISPR-CAS9-mediated gene editing. A homozygous G-to-A transition transformed codon 87 from alanine to threonine. Of note, the homozygous C-to-T transition in codon 89 was synonymous, which was performed to avoid gRNA-mediated secondary cleavage. **(B)** Validation of substitution of Ala87 by threonine in *Her2* p.A87T knock-in mice by Sanger sequencing. **(C)** Partial embryonic and preweaning lethality in *Her2* p.A87T knock-in mice. While *Her2*$^{A87T/A87T}$ embryos were observed at the expected Mendelian ratio (25%) at E13.5, their numbers declined markedly by E18.5 and P21. Mating schemes and genotypes used in the crosses were listed.

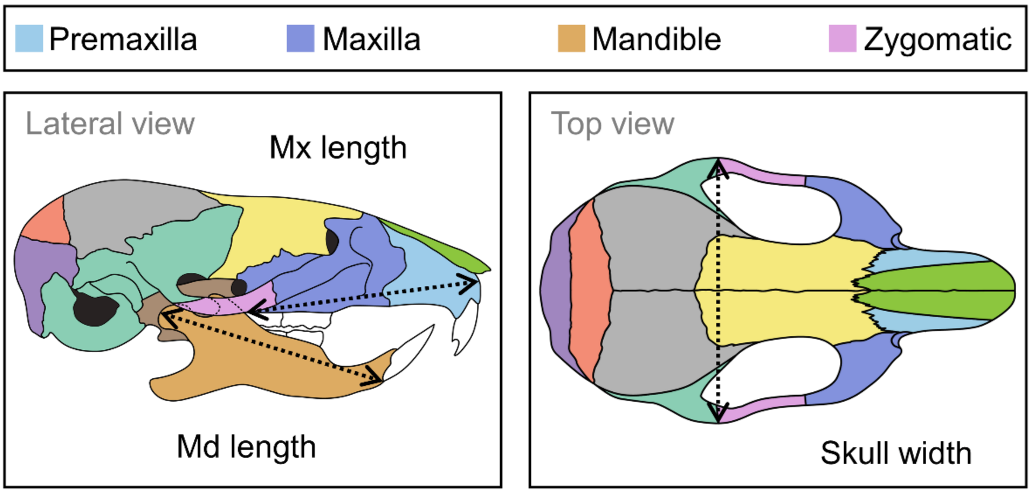

Supplemental Figure 6. Schematic diagram of mouse craniofacial bones, illustrating measurements of maxillary length, mandibular length, and skull width. Mx, Maxilla; Md, Mandible. Related to Figure 4F and Figure 5H.

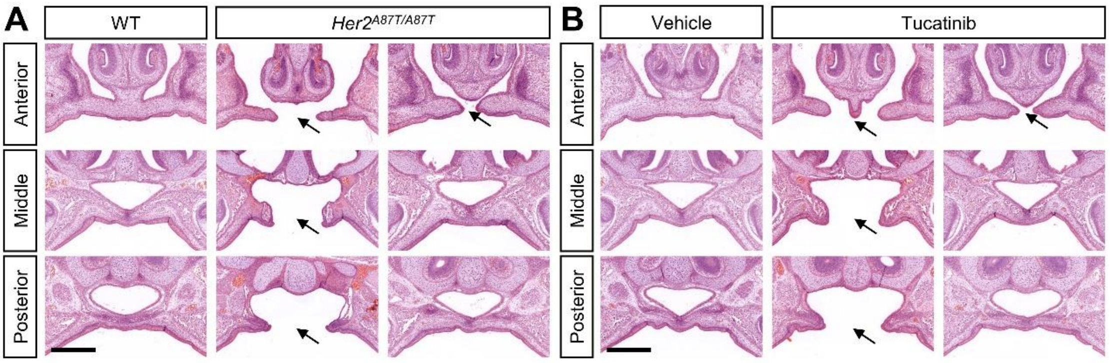

Supplemental Figure 7. Hematoxylin and eosin-stained coronal sections of E18.5 mouse heads reveal cleft palate phenotypes. Sections from anterior, middle, and posterior regions are shown for wild-type versus *Her2*$^{A87T/A87T}$ embryos (**A**; related to Figure **4G**, **H**) and vehicle- versus Tucatinib-treated embryos (**B**; related to Figure **5I**). Arrows indicate complete (middle column) or incomplete (right column) cleft palate. Scale bar: 500 µm.

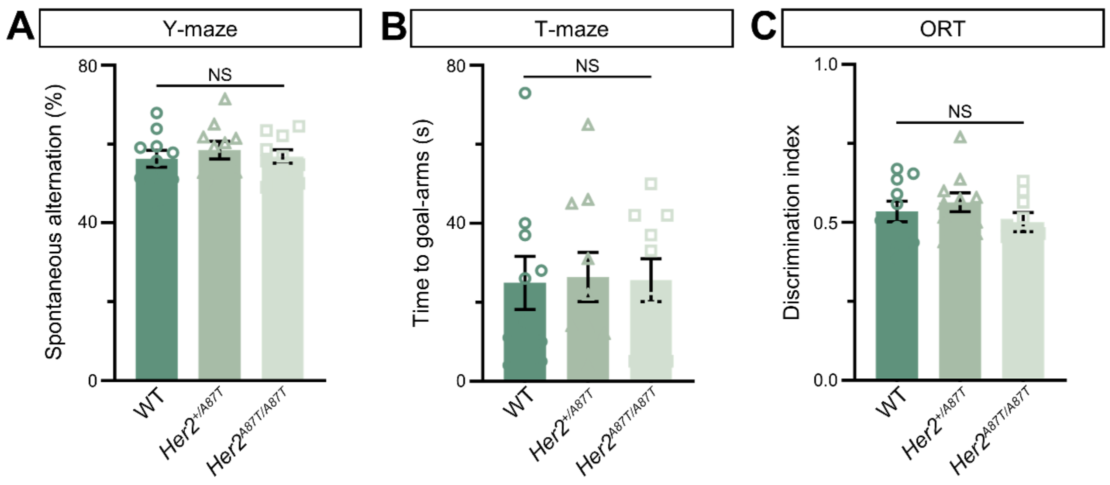

Supplemental Figure 8. WT, *Her2$^{+/A87T}$*, and *Her2$^{A87T/A87T}$* mice exhibit comparable cognitive-behavioral performance. Spontaneous alternation in the Y-maze test **(A)**, time to reach the goal arm in the T-maze test **(B)**, and discrimination index in the novel object recognition test (ORT) **(C)** are shown. Nine- to ten-week-old male WT, heterozygous (*Her2$^{+/A87T}$*), and homozygous (*Her2$^{A87T/A87T}$*) mice were subjected to cognitive behavioral assays (n ≥ 9 per genotype). In the ORT, one mouse was excluded due to insufficient total object exploration time (< 20 s). NS, not significant. Kruskal-Wallis tests (**A** and **C**) and one-way ANOVA (**B**).

Primer name	Primer sequence (5'-3')	Amplicon size	Variant
A87T-F	GACGGAGGTTGTGGTG	556 bp	p.A87T
A87T-R	TGACAGGGGTGGTATTG		
G603S-F	GCCTTGGGAAGCACA	337 bp	p.G603S
G603S-R	CCCCTTTTATAGTAAGAGCC		
R970W-F	GCACCACTGAAATCCAGCC	484 bp	p.R970W
R970W-R	AGCATGCAGCCTTCCG		
S1151W-F	CCTAATGGGTCACCTTCT	199 bp	p.S1151W
S1151W-R	GGTTCTCCACGGCAC		
T1242M-F	AAGAATGGGGTCGTCA	408 bp	p.T1242M
T1242M-R	CATCTGGGAACTCAAGC		

Supplemental Table 1. Primers used for PCR-Sanger sequencing to detect the p.A87T, p.G603S, p.R970W, p.S1151W, and p.T1242M variants in the human *HER2* gene.

Variants	ACMG classification	Evidence of pathogenicity
p.A87T	Pathogenic	PS3 + PS4 + PP1 + PP3
p.G603S	Pathogenic	PS2 + PS3 + PS4 + PP3
p.R970W	Pathogenic	PS2 + PS3 + PS4 + PP3
p.S1151W	Pathogenic	PS2 + PS3 + PM2 + PP3
p.T1242M	Pathogenic	PS3 + PS4 + PP1 + PP3

Supplemental Table 2. ACMG classification of p.A87T, p.G603S, p.R970W, p.S1151W, and p.T1242M variants in the human *HER2* gene. PS2: *De novo* variants confirmed with verified maternity and paternity; PS3: Functional studies *in vitro* and *in vivo* supporting the damaging effect on the gene; PS4: The prevalence of the variant in affected individuals is increased compared with controls (refer to OR in Table 1); PM2: Variant absent from controls in various databases; PP1: Co-segregation with disease observed in multiple affected family members; PP3: Multiple lines of computational evidence support a deleterious effect.
