# Supplementary material for: HER2 deficiency causes a developmental disorder with growth retardation and craniofacial malformations
Source: J Clin Invest. 2026 Apr 30;136(12):e199043. doi: 10.1172/JCI199043 (PMC13262720; doi:10.1172/JCI199043)

The original blot images

Figure 2D

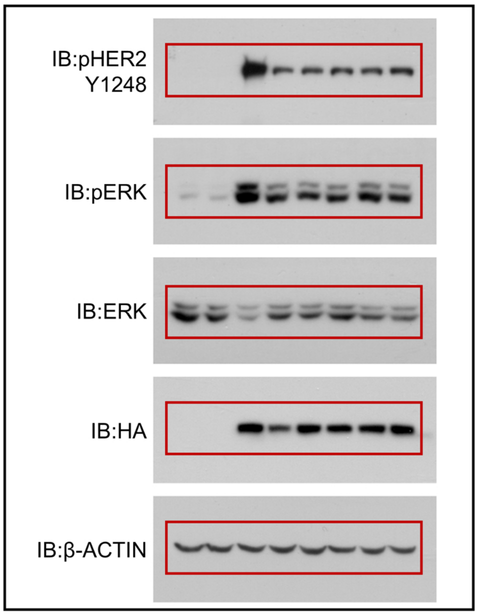

Figure 2F

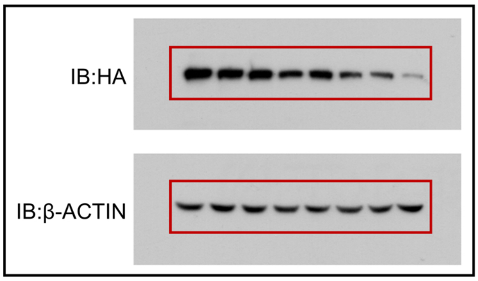

Figure 2G

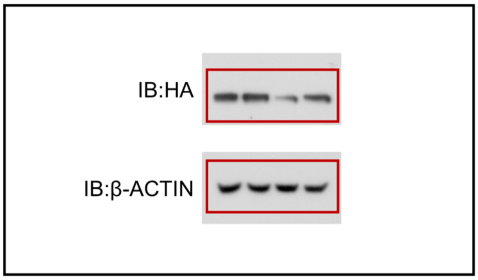

Figure 2H

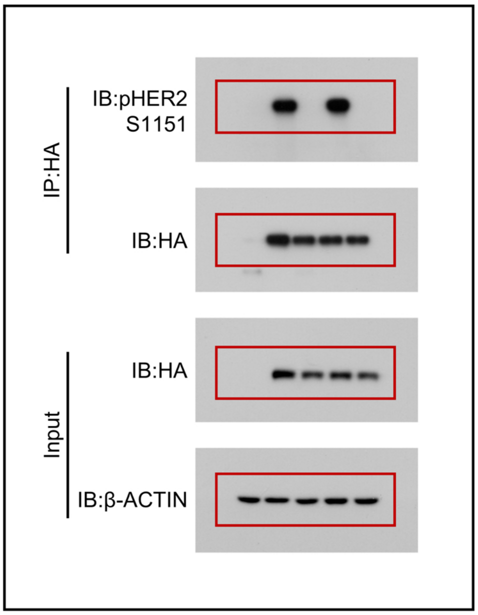

Figure 2I

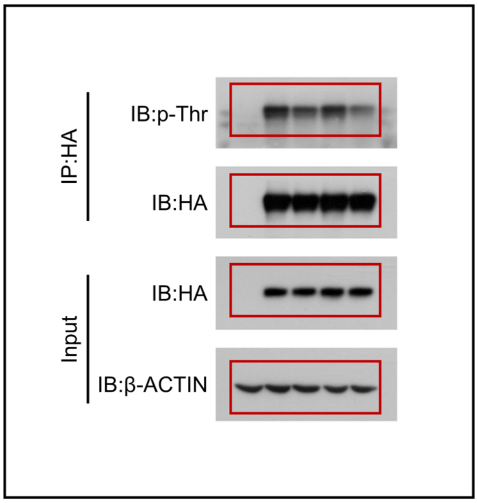

Figure 4J

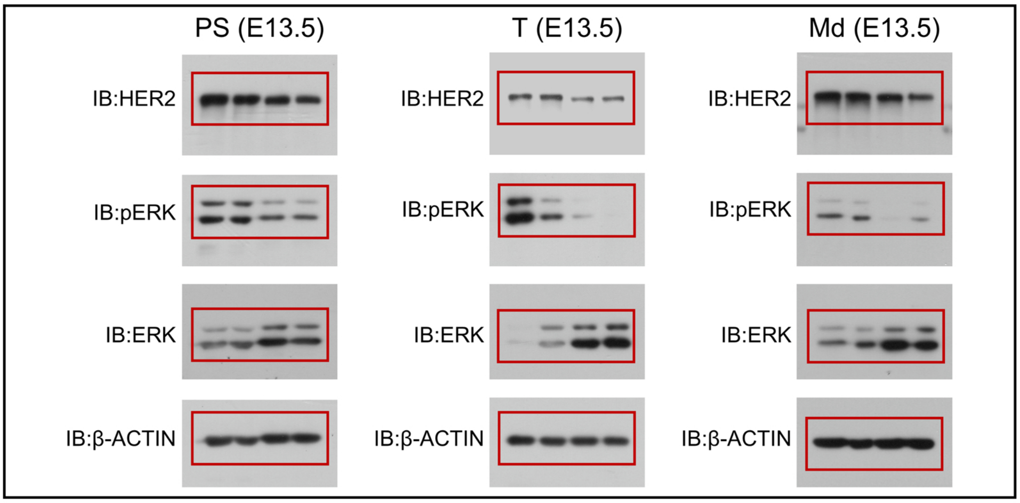

Figure 5B

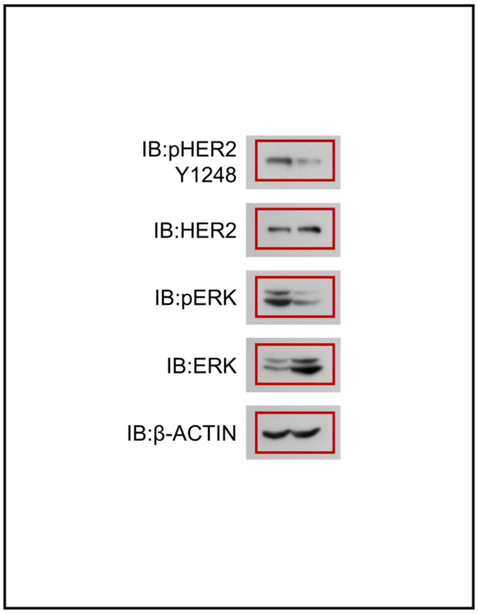

Figure S3A

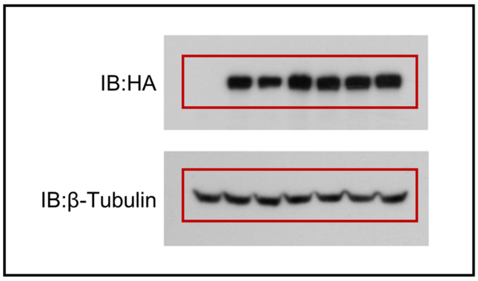

Figure S4C

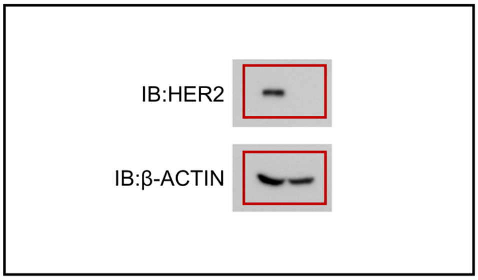

Figure S4D

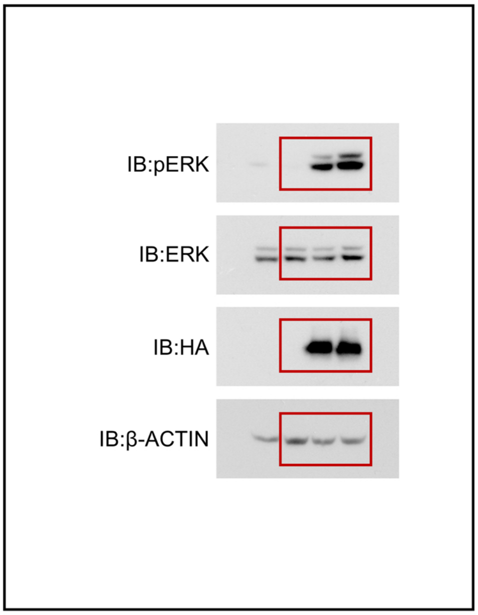

Supplement: Unedited blot and gel images [file jci-136-199043-s197.pdf]
